# Combined Treatment with an Oncolytic Adenovirus and Antitumor Activity of Vincristine against Retinoblastoma Cells

**DOI:** 10.3390/ijms130910736

**Published:** 2012-08-27

**Authors:** Xin Song, Haibo Wang, Renbing Jia, Biyun Cun, Xiaoping Zhao, Yixiong Zhou, Xiaofang Xu, Guanxiang Qian, Shengfang Ge, Xianqun Fan

**Affiliations:** 1 Department of Ophthalmology, Ninth People’s Hospital, Shanghai Jiao Tong University School of Medicine, Shanghai 200011, China; E-Mails: songxin0521@hotmail.com (X.S.); jrb6@sina.com (R.J.); catherine1_2_3@163.com (B.C.); zxp0856@sina.com (X.Z.); zhouyixiong21@gmail.com (Y.Z.); xuxu0139@hotmail.com (X.X.); 2 Department of Biochemistry and Molecular Biology, Shanghai Jiao Tong University School of Medicine, Shanghai 200025, China; E-Mails: wanghaibode@gmail.com (H.W.); qiangx@shsmu.edu.cn (G.Q.)

**Keywords:** retinoblastoma, vincristine, oncolytic adenovirus, Akt, drug resistance

## Abstract

Treatment trends of retinoblastoma (RB) have gradually evolved from eye enucleation and external radiation to local treatment. Combined treatment with an oncolytic virus and chemotherapy is currently a new method in RB treatment. To investigate the therapeutic effect of oncolytic adenovirus SG600 in combination with vincristine (VCR) on retinoblastoma *in vitro*, the cell viability, cell cycle effects and apoptotic activity of HXO-RB_44_ cells treated with SG600, VCR or SG600 plus VCR were measured using a cell counting kit-8-based procedure and flow cytometry. Western blot analysis for Akt, p-Akt, p-p53 and p-Rb protein was performed to investigate the underlying mechanisms of combined therapy. The combination therapy exerted a synergistic antitumor effect via a type of G_2_/M and S phase arrest rather than the induction of apoptosis. The combination of VCR and SG600 further reduced Akt phosphorylation compared with cells treated with VCR alone, suggesting that SG600 could overcome chemoresistance, perhaps by down-regulating Akt in RB cells. An increase in the expression of p-p53 and decrease in p-Rb expression in HXO-RB_44_ after co-treatment might be associated with cell cycle block. Western blot examination revealed that VCR might enhance SG600 replication. These results suggest that viro-chemo combination therapy is a feasible and potentially promising approach for the treatment of retinoblastoma.

## 1. Introduction

Retinoblastoma (RB) is the most common primary intraocular malignant tumor of childhood [1]. In recent years, with progress in the early detection and diagnosis of RB, treatment trends have gradually evolved from eye enucleation and external radiation to local treatment. Chemotherapy is commonly used to reduce tumor size before administration of local therapy. Conventional chemotherapy, although effective, lacks selectivity for tumor cells, and as a result often causes vomiting, fever, diarrhea, bone marrow suppression and leukemia as well as other side effects [2]. This problem is also associated with RB chemotherapy. Local injection of chemotherapeutic drugs may avoid the side effects of systemic chemotherapy, but problems of drug resistance still limit their application in clinical practice. A clinical study found that subconjunctival injection of cisplatin can cause significant weakening of RB, but this treatment is not ideal as it takes three to four weeks to take effect [3]. Chan *et al*. [4,5] first found that *P*-glycoprotein (*P*-gp) was highly expressed in RB cells after chemotherapy compared with untreated RB cells, suggesting that *P*-gp may be one of the main causes of drug resistance. Subsequently, Wilson *et al*. [6,7] have also reported that *P*-gp is associated with drug resistance of RB after chemotherapy. In view of the present situation regarding chemotherapy side effects and drug resistance, we hope to be able to find a new way to enhance the efficacy of chemotherapy drugs and reduce their side effects.

Combined treatment with an oncolytic virus and chemotherapy is a new method currently under investigation that has demonstrated encouraging results in refractory malignancies [8]. H101 oncolytic adenovirus is a human type 5 adenovirus, which has a manually deleted partial *E1B* region. H101 cannot replicate in normal cells; however, in tumor cells with *p53* gene mutations H101 can undergo unlimited replication, leading to tumor cell lysis and death, and the release of the virus which then continues to infect adjacent tumor cells, further expanding the killing effect. We showed in our previous experiments that HXO-RB_44_ cells treated with H101 had reduced cell viability. Abundant replication of H101 in HXO-RB_44_ cells resulted in G_2_/M phase arrest and finally tumor cell lysis, but the apoptosis pathway was not activated. Tumor-bearing mice treated with H101 had reduced tumor burdens and prolonged survival times [9,10].

Delta-24, a mutant adenovirus, which encodes an E1A protein with deletion of amino acids 120–127 that selectively targets cells with abnormal RB control, can replicate in cancer cells that have disrupted RB function. Thus, this virus can kill target cells, and amplify and spread the effect from cell to cell within the tumor, but does not affect the surrounding differentiated cells [11,12].

SG600 adenovirus has the characteristics of both H101 and Delta-24. In SG600, the CR2 region of the *EIA* gene is partly deleted, and the *E1A* and *E1B* genes are controlled by the hTERT promoter and the HRE, respectively. These modifications are expected to increase the capacity of viral replication and oncolysis, specifically targeting cancer cells and to decrease the viral cytotoxicity to normal cells [13]. An abnormal retinoblastoma susceptibility gene (RB1) is the main cause of the occurrence of RB. A deletion or point mutation of the RB1 gene can be detected in 80% of RB patients [14]. In retinoblastomas, the p53 pathway is frequently inactivated by amplification of MDMX and MDM2, which inhibit p53 activity by multiple mechanisms [15–17]. Due to the RB tumor characteristics mentioned above, RB could be a good target of SG600.

This study aimed to investigate the therapeutic effect on retinoblastoma of combined treatment with SG600 and vincristine (VCR), and to explore the possible mechanisms from the viewpoint of RB chemotherapy resistance and oncolytic adenovirus activity.

## 2. Results and Discussion

### 2.1. Cytotoxicity of HXO-RB_44_ Cells by the Combined Treatment of SG600 and VCR

**Figure 1:**
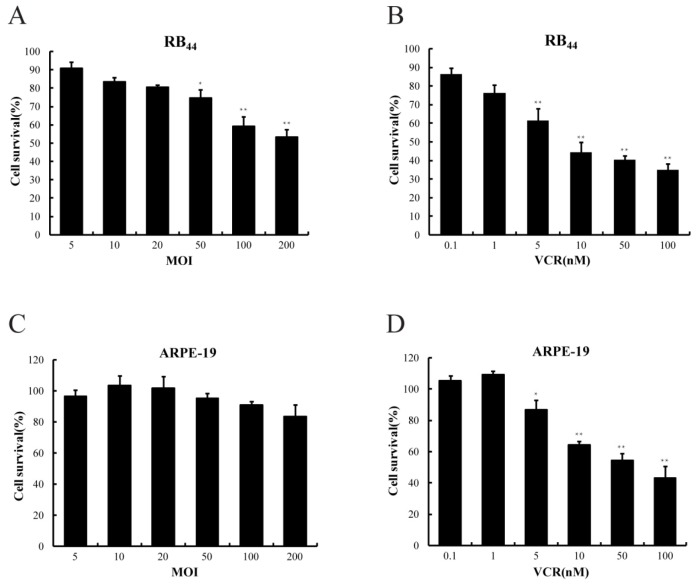
Growth inhibition following treatment with adenovirus (SG600) or vincristine (VCR) on HXO-RB_44_ (**A**,**B**) cells and normal cells (**C**,**D**). Bars: standard deviation. The results are representative of three independent experiments and of four replicates in each experiment. ** *p* < 0.01 relative to the SG600 or VCR treatment group.

**Figure 2:**
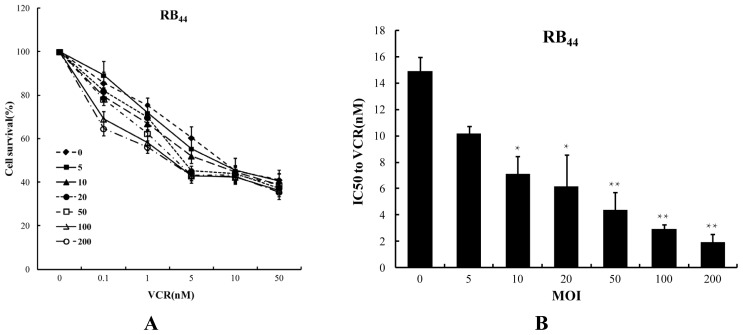
Cytotoxicity of VCR on HXO-RB_44_ cells with or without addition of adenovirus (SG600). (**A**) Cell viability of HXO-RB_44_ under different treatments; (**B**) for HXO-RB_44_ cells, IC_50_ values for VCR were changed in the different SG600 treatment groups. The results are representative of three independent experiments and of four replicates in each experiment. * *p* < 0.05, ** *p* < 0.01 relative to the SG600 (MOI = 0) treatment group. IC_50_: concentration of drug that is lethal for 50% of cells.

To examine whether the combination of SG600 and VCR enhances the antitumor effect of either agent alone in an RB cell line, HXO-RB_44_ cells were exposed to SG600 alone, VCR alone, and SG600 plus VCR. Cell toxicity, measured by the CCK-8 assay, was determined at 96 h after treatment (Figure 1 and 2A). As shown in Figure 1A,B, with increasing doses of SG600 or VCR, the survival rates of HXO-RB_44_ cells gradually decreased (*p* < 0.05) compared with the PBS group (NC). In contrast, the survival rates of ARPE-19 cells did not change significantly (Figure 1C) at the same MOIs of SG600, suggesting that SG600 had no significant killing effect on normal cells; however at the same doses of VCR, the survival rates of ARPE-19 were also affected (Figure 1D), showing the side effect of chemotherapy. We then studied the effect of combined treatment with SG600 and VCR. As shown in Figure 2A, compared with the treatment with SG600 or VCR alone, the survival rates of RB cells decreased markedly when treated with the combination of SG600 and VCR in the process with increasing dose. The survival rates of RB cells decreased from 60.48% ± 5.1% (5 nM VCR alone) to 45.12% ± 2.3% (20 MOI SG600 combined with 5 nM VCR) and 43.25% ± 2.4% (50 MOI SG600 combined with 5 nM VCR). With further increase in the dose of VCR (≥10 nM), the survival rates of RB cells treated with the combination of SG600 and VCR did not decrease significantly compared with the treatment with SG600 or VCR alone. Therefore, the dose of VCR was used with 5 nM in subsequent experiments. In another retinoblastoma cell line (WERI-Rb-1) similar results were also observed (data not shown).

Cytotoxicity of VCR in the HXO-RB_44_ cell line was also evaluated by IC50 values (Figure 2B). IC_50_ values for VCR decreased from 14.90 ± 1.03 nM (control, without SG600) to 7.12 ± 1.34 nM for 10 MOI SG600 (*p* < 0.05), to 4.38 ± 1.32 nM for 50 MOI SG600 (*p* < 0.01). These findings taken together indicate that combination treatment could achieve better cancer-killing effects with reduced drug toxicity.

### 2.2. Alteration of Cell Cycle Distribution by Combination Treatment

**Figure 3:**
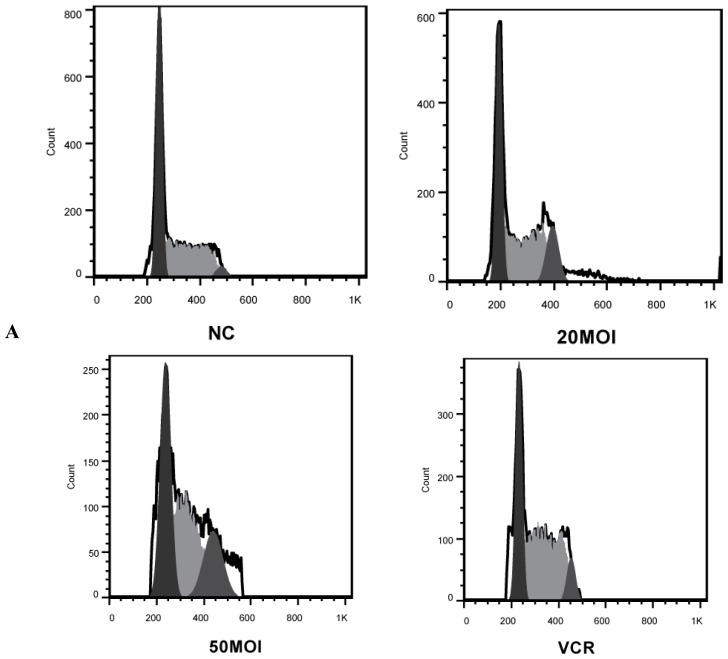

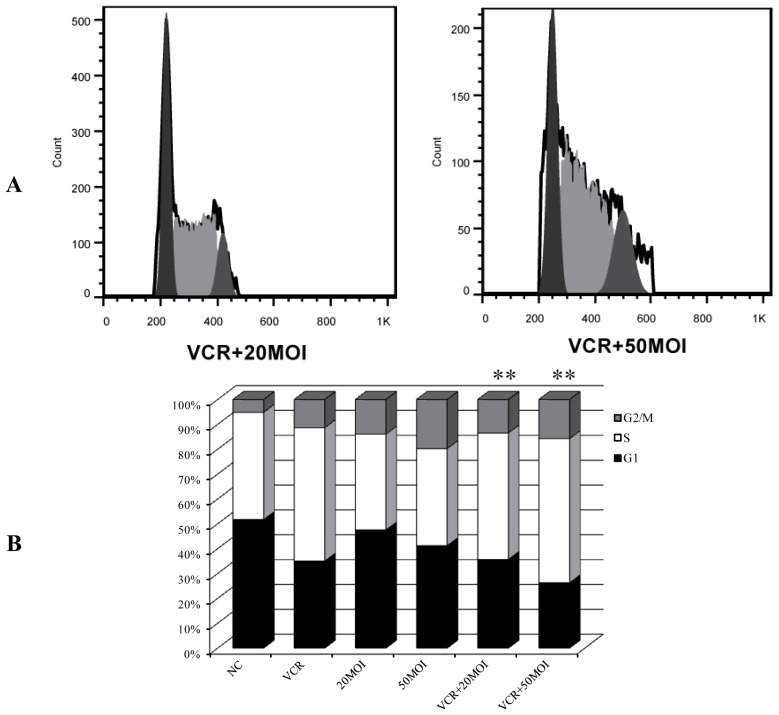
Representative FACS histograms showing the cell cycle distribution in HXO-RB_44_ cells following different treatments. (**A**) Cell cycle analysis was performed by quantifying propidium iodide (PI) incorporation by flow cytometry. DNA content and number of events were analyzed after different treatments for 48 h; (**B**) relative changes in the percentage in each cell cycle phase were plotted after PI staining and FACS analysis. Results are representative of three independent experiments (** *p* < 0.01, compared with S and G_2_/M phases of HXO-RB_44_ treated with 5 nM VCR). NC (negative control): treatment with PBS.

We next investigated whether our treatment scheme would affect the cell cycle. Following PI staining, cell cycle distribution analysis of HXO-RB_44_ cells was conducted 48 h later by flow cytometry. As shown in Figure 3, VCR (5 nM) led to increased cell cycle arrest in the S phase at 48 h post-treatment, whereas SG600 induced cell cycle arrest in the G_2_/M phase at 48 h post-treatment. In the co-treatment group, the cell cycle distribution exhibited a combination of the findings seen in the two monotherapy groups, with a moderate increase in the G_2_/M phase compared with the group treated with VCR (** *p* < 0.01).

### 2.3. Function of Apoptosis by Combined SG600 and VCR

**Figure 4:**
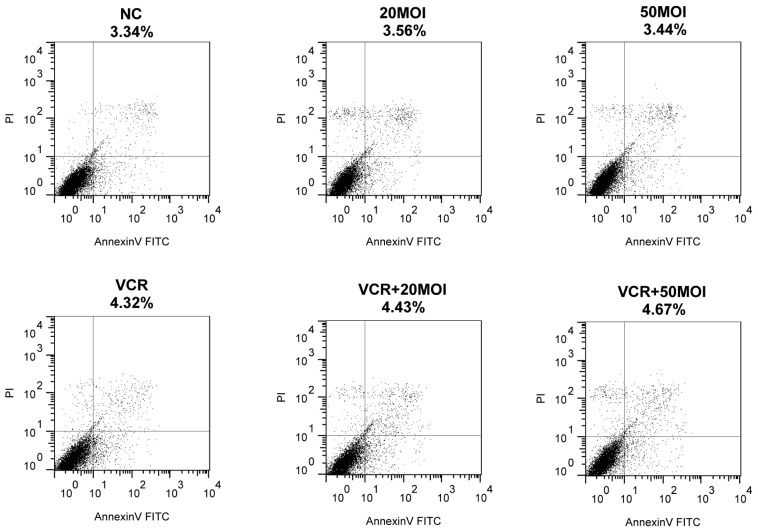
Detection of apoptosis in HXO-RB_44_ cells. Apoptosis was measured by flow cytometry analysis 48 h after treatment with VCR (5 nM), SG600, and VCR plus SG600. Results are representative of three independent experiments. NC (negative control): treatment with PBS.

To investigate the underlying mechanism of combination treatment, we analyzed apoptosis of HXO-RB_44_ cells when exposed to the various therapies. Forty-eight hours after treatment, apoptosis was assessed using flow cytometry and an Annexin V-FITC apoptosis kit. As seen in Figure 4, monotherapy with 5 nM VCR (4.32%) or SG600 (3.44%–3.56%) as well as combination treatment (4.43%–4.67%) did not induce a high level of apoptosis compared with the PBS control (3.34%).

### 2.4. P-Akt Protein Expression in HXO-RB_44_ Cells Following Combined Treatment with SG600 and VCR

**Figure 5:**
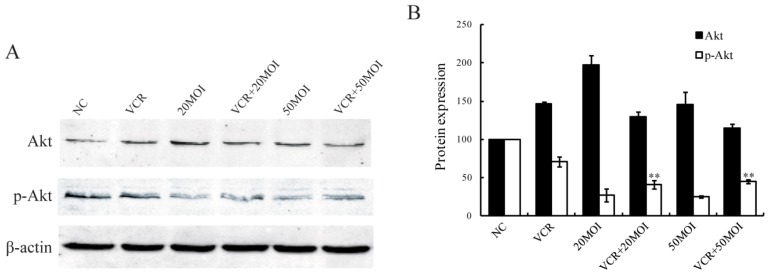
The expression patterns of Akt and phospho-Akt in HXO-RB_44_ cells after different treatments for 48 h. (**A**) The proteins were analyzed by immunoblot with specific antibodies; (**B**) Average band density of quantified Akt and phospho-Akt protein after normalization to the internal control β-actin. Protein expression of Akt and phospho-Akt in the NC group was arbitrarily set as 100. ** *p* < 0.01 relative to phospho-Akt protein expression in the 5 nM VCR-treated group. NC (negative control): treatment with PBS.

In HXO-RB_44_ cells, Akt phosphorylation was reduced after treatment with SG600 alone (*p* < 0.05). In addition, exposure of the cells to 5 nM VCR alone resulted in a decrease of Akt phosphorylation compared to control cells (*p* < 0.05). The combination of VCR + SG600 at the given concentration further reduced Akt phosphorylation compared with cells treated with 5 nM VCR alone (*p* < 0.01, Figure 5).

### 2.5. Effect of SG600 Replication by Combined SG600 and VCR

**Figure 6:**
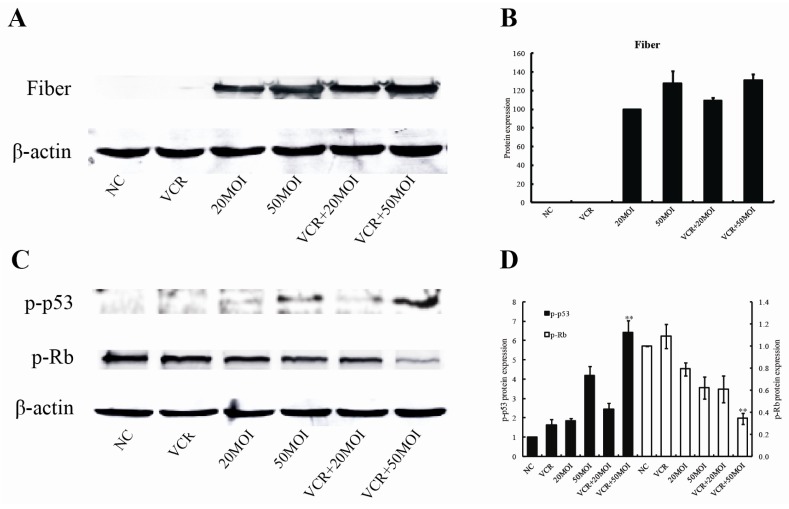
The expression patterns of Fiber, phospho-p53 and phospho-Rb in HXO-RB_44_ cells after different treatments for 48 h. (**A**) Western blot analysis of Fiber protein expression at 48 h; (**B**) Average band density of quantified Fiber protein after normalization to the internal control β-actin. Protein expression of Fiber in the SG600 (20 MOI) treatment group was arbitrarily set as 100; (**C**) western blot analysis of p-p53 and p-Rb protein expression at 48 h; (**D**) average band density of quantified p-p53 and p-Rb protein after normalization to the internal control β-actin. Protein expression of p-p53 and p-Rb in the NC group was arbitrarily set as 1. ** *p* < 0.01 relative to p-p53 and p-Rb protein expression in the SG600 or VCR (5 nM) treatment group. NC (negative control): treatment with PBS.

To evaluate whether VCR has any effect on the replication of SG600, we treated cells with SG600 (20 MOI or 50 MOI) alone, VCR (5 nM) alone, and SG600 plus VCR for 48 h, and then examined the level of adenoviral Fiber gene expression. In comparison to groups treated with SG600, the expression of Fiber in cells treated with SG600 combined with VCR was slightly increased, although it did not reach statistical significance (*p* > 0.05, Figure 6A,B). The results indicated that VCR might not interfere with SG600 replication.

### 2.6. Altered p-p53 and p-Rb Protein Expression Following Combined Treatment with SG600 and VCR

We treated cells with SG600 alone, VCR alone, and SG600 plus VCR for 48 h, and then examined the level of p-p53 and p-Rb proteins. As shown in Figure 6C,D, in comparison to groups treated with VCR (5 nM) alone (p-p53: 1.62, p-Rb: 1.08) and SG600 (50 MOI) alone (p-p53: 4.19, p-Rb: 0.62), the expression of p-p53 protein (6.41) was significantly increased in the VCR (5 nM) plus SG600 (50 MOI) treatment group (*p* < 0.01), and at the same time the expression of p-Rb protein (0.35) was significantly reduced (*p* < 0.01).

### 2.7. Discussion

Combined treatment with an oncolytic adenovirus and chemotherapy presents a promising novel treatment strategy for cancer and is rapidly advancing toward clinical use in many malignancies [18–21]. In the present study we investigated the therapeutic effect of the oncolytic adenovirus SG600 in combination with the chemotherapeutic drug VCR in the treatment of RB cell lines.

Chemotherapy has recently achieved a major role in the primary management of intraocular retinoblastoma. Recent experience has proved that use of chemotherapy for intraocular retinoblastoma before local treatment (so called “chemoreduction”) has been accepted as treatment strategy with the goal of avoiding external beam radiotherapy (EBRT) or enucleation [22]. As one of the common treatments, VEC (vincristine, etoposide, carboplatin) chemotherapy combined with delayed local therapy (cryotherapy, photocoagulation, brachytherapy) has allowed not only to decrease number of enucleations and indications for external beam irradiation or limit the extension of local therapy, but also increase chances for vision preservation and decrease the risk of severe complications [23,24]. VCR is a first line chemotherapeutic agent for RB, but its application is affected by side effects and drug resistance [25–27]. In agreement with its reported effects, our results also show that the killing effect on HXO-RB_44_ cells was enhanced with increasing VCR dosage, but the damage to ARPE-19 cells was increased at the same time. VCR is not able to kill cancer cells selectively (Figure 1B,D). In contrast, virotherapy with CRAds is based on their cancer selectivity by confining viral replication within cancer cells. SG600 was modified by controlling the *E1A* gene with the hTERT promoter and the *E1B* gene with the HRE to improve the safety of this CRAd [13]. The hTERT and HRE promoters are found in most human solid tumors due to the existence of hypoxic areas in the tumor tissues and the expression of telomerase in the cancer cells.

In the case of RB, hypoxia is an essential feature and contributes to poor prognosis and resistance to conventional therapy. That the virus containing the hTERT promoter can play a role in RB treatment has also been proven [28]. Both *E1A* and *E1B* genes are necessary for efficient viral replication. E1A interacts with RB and E1B interacts with p53, which can affect viral replication. These features mean that SG600 can selectively kill RB cells, while having no effect on normal cells (Figure 1A,C). As shown in Figure 2A, synergistic antitumor activity was observed following combination treatment. The IC_50_ of VCR decreased after combination treatment, indicating that the sensitivity of RB cells to VCR was increased (Figure 2B).

The precise mechanism of this enhanced activity of combined VCR therapy and viral oncolysis is still unclear. Our investigation demonstrated that tumor cell death had little to do with apoptosis, whether through monotherapy of VCR or SG600, or through co-treatment, which appears to be related to cell cycle blocking (Figures 3 and 4). SG600 can induce G_2_/M phase block on RB while H101 has the same function. VCR induced S-phase block in RB cells. The combination of treatment induced both G_2_/M and S phase arrest.

It is also interesting to note that expression of both p53 and p-Rb were changed after combination treatment. As is well known, p53 is one of the most important tumor suppressor genes. It is estimated that the p53 pathway is inactivated in 75% of retinoblastoma patients [16]. The retinoblastoma gene is a tumor suppressor gene that codes for the Rb protein [29]. RB occurs if any mutation inactivates both normal alleles. The Rb protein is a regulator at the cell cycle checkpoint between G1 and entry into the S-phase. The phosphorylation pattern of p-Rb varies during the cell cycle and the current model suggests that the hypophosphorylated normal p-Rb binds transcriptional regulators that promote entry into the S-phase. This function of p-Rb is inactivated by phosphorylation or by viral oncoprotein binding of p-Rb. When phosphorylated, p-Rb dissociates from the transcription factor E2F, freeing E2F to bind to DNA and stimulate transcription of downstream genes that promote progression through the cell cycle. Loss of normal RBl function, as in the case of tumors, presumably allows for uncontrolled entry into S-phase and more rapid cell division [30,31]. As shown in Figure 6C,D, in comparison to groups treated with VCR alone or SG600 alone, the expression of p-p53 protein was significantly increased in the VCR plus SG600 (50 MOI) treatment group (*p* < 0.01), and at the same time the expression of p-Rb protein was significantly reduced (*p* < 0.01). This may be the reason why S phase and G_2_/M phase arrest is induced by combination therapy in RB cells.

Akt (protein kinase B, PKB) is a serine/threonine kinase, which is a central regulator of many cellular processes including proliferation, differentiation, survival, and metabolism [32,33]. It has been previously reported that Akt activity was involved in VCR-induced cytotoxicity in retinoblastoma SO-Rb50 cells. Inhibition of Akt can reduce the survival of SO-Rb50 cells and increase the sensitivity of cell lines to VCR [34]. We found that the combination of VCR and SG600 at the given concentration further reduced Akt phosphorylation compared with cells treated with VCR alone, suggested that SG600 overcomes chemoresistance, perhaps by down-regulating Akt in RB cells.

It has been reported that some drugs may enhance adenovirus replication when added after virus infection [35]. We tested whether VCR interfered with replication of adenovirus SG600. VCR affects expression of the late gene Fiber, as detected by Western blot (Figure 6A), indicating that VCR may not interfere with SG600 replication and even slightly enhance the killing ability of SG600 through up-regulation of virus replication.

These results suggest that viro-chemo combination therapy is a feasible and potentially promising approach for the treatment of retinoblastoma. Although our results are encouraging, our study had some limitations. First, it was a cell-culture study in which the cells had an artificial environment and it was not a real clinical condition. Second, we cannot rule out the possibility that other factors besides cell cycle and apoptosis, such as autophagy, antiangiogenic and immunosuppressant effects, could be contributing to the synergy observed in the viro-chemo combined treatment. Third, the mechanisms by which the combined treatment exerts its effect on the tumor cells are not all clear yet. Therefore, further studies about the mechanisms of viro-chemo combination therapy *in vivo* are necessary.

## 3. Experimental Section

### 3.1. Chemicals and Reagents

Cell counting kit-8 (CCK-8) was from Dojindo Laboratories. RPMI 1640 medium was from Gibco BRL (Gaithersburg, MD, USA). Anti-phospho-Akt (Ser473) and anti-Akt antibodies were from Bioworld (Louis Park, MN, USA), anti-phospho-p53, anti-phospho-Rb, and β-actin antibodies were from Cell Signaling Technology (Danvers, MA, USA), anti-Fiber antibody was from Abcam (Cambridge, MA, USA). Fluorescent-labeled secondary antibodies were from Rockland (Gilbertsville, PA, USA). Vincristine (VCR) was from Selleck Chemicals LLC (Houston, TX, USA).

### 3.2. Oncolytic Adenoviruses

SG600 was a gift from Professor Qi-Jun Qian, Laboratory of Viral and Gene Therapy, Eastern Hepatobiliary Surgery Hospital, Second Military Medical University (Shanghai, China). SG600 is a replication competent adenovirus with deletion of the *E1A* promoter, the *E1B* promoter, and the 24 nucleotides of the adenovirus *E1A* CR2 region.

### 3.3. Cell Lines and Cell Culture

The human retinoblastoma cell line HXO-RB_44_ (kindly provided by Heping Xu, Central South University, Changsha, China) was cultured in RPMI 1640 supplemented with 10% fetal bovine serum (FBS). The human retinal pigment epithelial cell line ARPE-19 (Kindly provided by the Department of Ophthalmology, Ruijin Hospital, Shanghai Jiaotong University School of Medicine, Shanghai, China) was cultured in DMEM/F-12 (1:1) supplemented with 10% FBS. All cell lines were maintained at 37 °C with 5% CO_2_.

### 3.4. Cell Viability Assay

HXO-RB_44_ and ARPE-19 cells were seeded into 96-well plates, incubated overnight then treated with VCR, SG600, or both agents in combination. Cell viability was quantified using a cell counting kit-8 (CCK-8; Dojindo, Kumamoto, Japan) at 96 h after infection as previously described [9]. The cytotoxicity of VCR in the cells was expressed as the IC_50_ value evaluated by the CCK-8 test. A drug resistance reversal effect was evaluated as a decrease in the IC50 for VCR induced by the presence of SG600. All data were obtained in triplicate.

### 3.5. Cell Cycle

HXO-RB_44_ cells were harvested at 48 h after treatment with VCR, SG600, a combination of both agents, or PBS as the negative control (NC), fixed in 100% chilled ethanol, and kept at −20 °C for at least 24 h. To measure DNA content, cells were washed twice with PBS and stained with 25 μg/mL propidium iodide (PI; Sigma-Aldrich, St Louis, MO, USA). Cell cycle distribution was determined by flow cytometry. Ten thousand events were acquired for each sample and analyzed.

### 3.6. Apoptotic Analysis

Apoptosis was analyzed by flow cytometry following dual staining with annexin-V-fluorescein isothiocyanate and PI. Cells were prepared according to the manufacturer’s instructions provided with the Annexin V-FITC apoptosis kit (BD Biosciences, San Jose, CA, USA). Apoptosis was quantified on a fluorescence-activated cell sorter (Becton Dickinson, Sunnyvale, CA, USA), and data from 10,000 events were collected for further analysis.

### 3.7. Western Blot Analysis for Akt, p-Akt, Fiber, p-p53 and p-Rb Protein

HXO-RB_44_ cells were harvested and used for protein extraction for Western blotting. Proteins were separated by sodium dodecyl sulfate–polyacrylamide gel electrophoresis in 12% (wt/vol) polyacrylamide gels and transferred to polyvinylidene fluoride (PVDF) membranes. Membranes were incubated with primary antibodies to Akt, p-Akt, Fiber, p-p53 and p-Rb, with β-actin as a loading control, then after washing they were incubated with a secondary antibody conjugated to a fluorescent tag; the bands were visualized and quantified by an infrared imaging system (Odyssey; LI-COR, Lincoln, NE, USA).

### 3.8. Statistical Analysis

All experiments were performed in triplicate, and data are expressed as mean ± SD. The data were analyzed with Student’s *t*-test, and results were considered statistically significant at *p* < 0.05.

## 4. Conclusions

In conclusion, our findings show that the combination of VCR and SG600 exerts an efficient RB cell killing effect, suggesting that this combination is potentially promising for the effective treatment of RB. However the underlying mechanisms still need to be elucidated in order to enhance the efficacy of virus-based gene therapy.
